# Modeling and analysis of infectious diseases based on behavioral game theory on two-layered networks under media coverage

**DOI:** 10.1371/journal.pone.0320904

**Published:** 2025-05-20

**Authors:** Jianrong Wang, Rong Zeng, Xinghua Chang

**Affiliations:** 1 School of Automation and Software Engineering, Shanxi University, Taiyuan, China; 2 School of Mathematics and Statistics, Shanxi University, Taiyuan, China; 3 Department of Mathematics, Taiyuan University, Taiyuan, China; Asansol Polytechnic, INDIA

## Abstract

The spread of infectious diseases poses significant threats to public health, the economy, and society as a whole. Despite governmental control measures over individual behavior, the public might still be influenced by factors such as costs, expected benefits, and the behavior of others, leading to incomplete adherence to disease control measures. Therefore, this paper proposes a behavioral game theory based infectious disease model on two-layer networks. First, considering the dynamic interaction between individual awareness behavior and disease spreading, a two-layer coupled network of individual behavioral awareness and disease spreading is established. Second, game theory is used to describe the impact of relevant factors on individual awareness behavior. The first layer represents the individual protective behavior game layer, while the second layer represents the disease spreading layer. Government intervention in individual behavior is also considered in the two-layer network model, according to the situation of infectious disease spreading, a threshold model is introduced to control individual protective behavior. Finally, MMCA is used to analyze the disease spreading threshold, and the proportion of the final population state and the spreading threshold under different model parameters are analyzed. The results show that by reducing personal protective costs, increasing individual attention to disease information, and enhancing governmental adjustments to disease control measures, the outbreak threshold of disease spreading can be effectively increased.

## 1 Introduction

Infectious diseases pose a serious threat not only to individual health but also to public health safety at the community and global levels [1, 2]. Therefore, many scholars have conducted research on the spread of infectious diseases. During the 2003 SARS outbreak, simple self-protection measures taken by individuals upon receiving SARS-related information, such as reducing travel, avoiding crowds, washing hands frequently, staying at home, and wearing masks, along with mandatory measures from governments or health departments, effectively controlled the spread of the disease [3–6]. Sambaturu *et al*. [7] developed strict approximation algorithms for single-stage and two-stage vaccine allocation to control the spread of epidemics through vaccine deployment. Venkatramanan *et al*. [8] integrated short-term and long-term population movement patterns into the SEIR model to study the spreading patterns of seasonal influenza and vaccine distribution issues. Brockmann *et al*. [9] replaced traditional geographic distance with probabilistic effective distance, establishing a homogeneous SIR model to predict the peak time of infections.

Complex network theory provides a framework and tools for modeling and analyzing the spread of diseases and information [10–12]. Researchers have extensively investigated the spread of diseases [13–15] and information [16–18] within complex network frameworks. Gross *et al*. [19] found that network structure significantly affects disease spreading. Many scholars have also started using multiplex networks as the underlying framework to study the dynamic interaction between information dissemination and disease spreading. Yin *et al*. [20] established a three-layer coupled network model to analyze the co-evolution of vaccine-related negative information, vaccination behavior, and disease spreading. Wang *et al*. [21] developed the UAU-SIR model to study the multiple impacts between awareness diffusion and disease spreading, finding that the disease threshold is related to the awareness diffusion and the network topology of the disease. Shi *et al*. [22] proposed the UAU-SEIS model to analyze the dynamic interaction between behavior and disease spreading in multiplex networks, considering the impact of individual heterogeneity on disease dynamics. Xia *et al*. [23] constructed a two-layer network model for the spread of information and epidemics on signed networks, assuming that information reception rates among individuals vary based on positive and negative relationships, and that individual prevention intensity varies with local and global disease proportions. Wu *et al*. [24] believed that individual perception of infection risk depends on local, global, and contact information, finding that local and contact information can increase the epidemic threshold. Huang *et al*. [25] studied whether people facing asymptomatic infections trust social influence or risk perception more, establishing a disease model in multiplex network topologies. Sun *et al*. [26] investigated the impact of resource diffusion on disease spreading in two-layer higher-order networks, showing that increasing resource dispersion on 2-simplexes can suppress disease spread and outbreaks. Li *et al*. [27] considered local and global information as well as individual differences, establishing a multi-information disease spreading model on two-layer networks. Guo *et al*. [28] studied the impact of awareness diffusion on disease outbreaks in multiplex networks, finding that community information affects the disease threshold in two stages, leading to different final disease scales. Xia *et al*. [29] proposed an SIQRS model with quarantine, studying its evolution on simplexes and using the QMF method to derive the spreading threshold and the steady-state infection ratio and stability conditions. Xie *et al*. [30] investigated the impact of heterogeneity in individual behavior and physiology on epidemic transmission, proposing two metapopulation SIR models from both individual and group perspectives and deriving the basic reproduction numbers for the models. Feng *et al*. [31] introduced an epidemic spreading model based on a two-layer network to describe the influence of individuals with different attributes in the awareness layer on epidemic dynamics, including those who ignore the epidemic. They analyzed how individuals with varying properties in the awareness layer affect the spread of disease.Fan *et al*. [32] studied an infectious disease model with a simple composite structure on multilayer networks, using the microscopic Markov chain method to derive probability transition equations and determine the epidemic outbreak threshold. Wang *et al*. [33] proposed a novel epidemic-awareness coupling model on multiplex networks, incorporating asymptomatic states and self-initiated awareness mechanisms to examine how these factors affect the dynamics of both epidemics and awareness.

However, existing research on disease modeling has some limitations, capturing overly simplified behaviors (e.g., whether to maintain social distance) and failing to capture the complex mechanisms behind behavioral responses, including individual perceptions of infection risk and bounded rationality, government orders, socio-economic costs, fatigue in complying with containment policies, and social influence [34–37]. Game theory models have been proven effective in reproducing decision mechanisms in complex scenarios, capturing behavior responses consistent with real-world scenarios across different fields [38]. Xia *et al*. [39] studied the impact of network topology on N-person trust games by considering second-order reputation rules, proposing a game model consisting of investors (trustors), trustworthy trustees, and untrustworthy trustees, exploring how degree distribution and clustering coefficients affect cooperative behavior through different social networks and defection temptation.Xiong *et al*. [40] explored how social relationships influence our tendencies toward specific individuals and the strength of pre-established relationships within the framework of the weak prisoner’s dilemma on multilayer networks. Zhang *et al*. [41] investigated a seasonal influenza-like disease model incorporating the game relationship between subsidy policies and human behavioral responses. Steinegger *et al*. [42] proposed and analyzed an imitation-driven mechanism at the individual level, considering perceived risk of infection and direct costs of protective behavior, showing that it could generate sustained stable oscillations. Chen *et al*. [43] studied how social imitation dynamics of vaccination are affected by imperfect vaccines. Ge *et al*. [44] proposed a mathematical model combining mandatory and voluntary vaccination, comparing the expected benefits and vaccination situations of different game strategies. Feng *et al*. [45] explored how changing cognition affects individual vaccination behavior and vaccine dynamics, proposing a model combining disease dynamics and evolutionary game theory.

This paper incorporates government intervention in individual behavior into a two-layer network model and models the game behavior among individuals under disease conditions. Considering the costs and benefits of personal protective behavior, the dynamic interaction between individual protective behavior and SEIR-based disease spreading is studied. The main contributions of this paper are as follows: (1) A behavioral transition model based on game theory is proposed to address individual trade-offs for protective behavior during disease spreading. (2) Government intervention in individual behavior is included in the two-layer network model, and a threshold model is introduced to control individual protective behavior according to the spread of the disease. (3) MMCA is used to analyze the disease spreading threshold, the proportion of the final population state and the spreading threshold under different model parameters are analyzed. The structure of the paper is as follows: Sect 2 introduces the MUM-SEIR model, Sect 3 uses MMCA to analyze the disease spreading threshold, Sect 4 presents simulation results, and Sect 5 concludes.

## 2 Model introduction

Government regulations on personal protective behavior during the spread of a disease are crucial for controlling its spreading. However, due to the influence of numerous complex factors such as different individual risk perceptions, disease situations, personal economic conditions, special needs, or social influence, it cannot be guaranteed that all individuals will comply with government-issued disease prevention rules.

**Fig 1:**
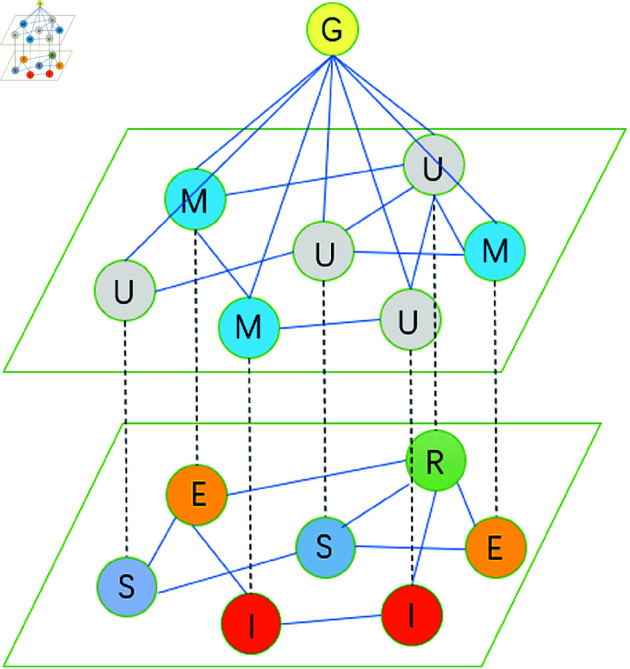
Schematic diagram of the disease model on a two-layer network.

Therefore, this paper considers the dynamic interaction between individual protective behavior and the spread of infectious diseases, establishing a two-layer coupled MUM-SEIR infectious disease model (as shown in Fig 1). The first layer is the individual behavioral awareness layer MUM, where *M* represents individuals adhering to disease prevention rules issued by the government, and *U* represents individuals who fail to fully comply with anti-disease rules (such as not wearing masks, not maintaining social distance, not getting vaccinated, not adhering to quarantine and self-isolation regulations, etc.). The second layer is the disease spreading layer SEIR, where *S* represents susceptible individuals, *E* represents exposed individuals, *I* represents infected individuals, and *R* represents individuals who have acquired immunity to the disease. At the same time, a government intervention layer for managing individual behavior is introduced. This layer represents the disease control measures and regulations formulated and issued by the government, which will adjust individual behavior according to the current disease situation, i.e., intervene in the MUM layer. Define *z*(*t*) represents the current global epidemic infection status, *m*_*i*_(*t*) as the strength of government control under the current disease situation, and *w*_*i*_(*t*) as the threshold function for government-regulated individual behavior, with the expressions as follows:

z(t)=(I(t)+E(t))/(S(t)+E(t)+I(t)+R(t)) (1)

$$m_{i}(t)=1/(1+e^{-\delta (z(t)-0.5)})$$ (2)

$$w_{i}(t)=H\left[\theta -z(t)\right]m_{i}(t)$$ (3)

where δ is the strength of the control measures, θ is the government intervention threshold. *H*(*x*) is the Heaviside step function, when x>0,H(x)=1; when x≤0,H(x)=0. *m*_*i*_(*t*) fluctuates with the value of *z*(*t*), indicating that individual control measures are weaker when the epidemic situation is less severe. *w*_*i*_(*t*) characterizes the control measures related to *z*(*t*). When the disease situation is below the threshold, meaning the government considers the current disease situation to be controllable, individual behavior is subject to the government’s normal disease control measures for individuals, and individuals in state *U* transition to state *M* with probability *w*_*i*_(*t*); When the disease situation exceeds the threshold, meaning the government considers the current disease situation to be severe, it will take stringent control measures, and all individuals in state *U* will be forcibly converted to state *M*.

**Fig 2:**
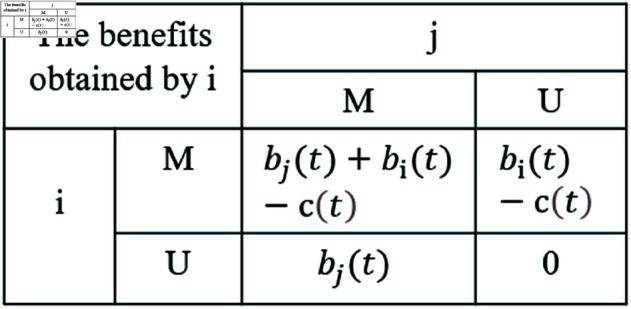
Behavioral game theory payoff matrix.

The modeling of individual behavioral games theory in the MUM layer is as follows: Considering individuals’ protective behaviors against infectious diseases (e.g., whether to get vaccinated, maintain social distance, wear masks, etc.), these decisions are influenced by personal costs, expected benefits, and the behavior of others. Therefore, game theory is used to describe the tendencies of individual behavior. Individuals choose different ways to respond to infectious diseases based on the current disease situation, personal needs, and so on. The payoff matrix is defined as shown in Fig 2:

$$b_{i}(t)=q_{i}^{M}(t)-q_{i}^{U}(t)$$ (4)

$$b_{j}(t)=q_{j}^{M}(t)-q_{j}^{U}(t)$$ (5)

$$q_{i}^{U}(t)=\prod_{j}\left[1-b_{ij}(p_{j}^{MI}(t)+p_{j}^{UI}(t)+p_{j}^{ME}(t)+p_{j}^{UE}(t))\beta^{U}\right]$$ (6)

$$q_{i}^{M}(t)=\prod_{j}\left[1-b_{ij}(p_{j}^{MI}(t)+p_{j}^{UI}(t)+p_{j}^{ME}(t)+p_{j}^{UE}(t))\beta^{M}\right]$$ (7)

$$c(t)=c+\sum_{\tau =1}^{t}a^{t-\tau }\varphi e^{-\frac{t}{\eta }}$$ (8)

where $q_{i}^{U}(t)$ and $q_{i}^{M}(t)$ represent the probabilities that nodes in states *M* and *U*, respectively, are not infected by their neighbors, *B* = (*b*_*ij*_) represent the adjacency matrices of the lower layers, $\beta^{U}$ and $\beta^{M}$ represent the infection rate under *U* and *M*, respectively. $p_{j}^{M\text{E}}(t),p_{j}^{M\text{I}}(t),p_{j}^{U\text{E}}(t)$ and $p_{j}^{U\text{I}}(t)$ represent the proportions of the corresponding states at time *t*. The benefits obtained by node *i* is described as the increase in the probability of not being infected when in contact with an individual, where *b*_*i*_(*t*) represents the increased probability of node *i* not being infected due to personal protection and adherence to government-issued infectious disease rules, and *b*_*j*_(*t*) represents the corresponding probability for neighbor node *j*. *c*(*t*) indicates the cost that changes over time, where c∈[0,1] represents the immediate cost, such as basic hygiene costs, psychological stress, and cumulative protection costs. a∈[0,1] represents the accumulation factor indicating the influence of past protective behavior on the current compliance cost. φ∈[0,1] indicates the scale of costs related to protective behavior, and $e^{\left(-t/\eta \right)}$ represents the fatigue function, with the compliance factor η∈[0,1] regulating the depiction of the public’s decreasing compliance with government control policies over time.

The total payoff *D*_*i*_(*t*) of node *i* is defined based on the states of its neighbors as follows:

$$D_{i}(t)={\sum \;\;}_{j\in Ni}b_{j}(t)\cdot s_{j}-c(t)\cdot s_{i}+b_{i}(t)\cdot s_{i}$$ (9)

where $j\in N_{i}$ represents the set of neighbors of node *i*, and *s*_*j*_ ,*s*_*i*_ represent the behavioral strategy choices of nodes *j* and *i* regarding states *M* and *U*, respectively. *s*_*i*_ = 1 indicates the individual chooses the *M* behavior, and *s*_*i*_ = 0 indicates the individual chooses the *U* behavior.

From the set of neighbors *N*_*i*_ of node *i* and the benefit calculation formula *D*_*i*_(*t*), the average benefit of the neighbors of node *i*, $\overset{―}{D_{i}^{j}}(t)$ is obtained as follows:

$$\overset{―}{D_{i}^{j}}(t)={\sum \;\;}_{j\in Ni}D_{j}(t)/j$$ (10)

Using the Fermi update rule, individual *i* will update their state with probability *F*_*i*_(*t*) based on their own and their neighbors’ benefits as follows:

$$F_{i}(t){=1/(1+e}^{-\frac{D_{i}\text{(t)\textasciitilde{}}-\overset{―}{D_{i}^{j}}(t)}{\omega }})$$ (11)

where ω represents the difference factor between *D*_*i*_(*t*) and $\overset{―}{D_{i}^{j}}(t)$. A larger ω indicates that the individual is less likely to change their behavior.

To represent the individual’s perception of the current global disease situation, the disease information perception function r(z(t)) is defined as follows:

$$r\left(z(t)\right)=\eta \cdot {z(t)}^{\alpha }$$ (12)

this function is regulated by η∈[0,1], where *z*(*t*) is the current disease prevalence rate, and α is the parameter representing the degree of importance placed on risk. α>1 indicates low importance placed on risk, while α<1 indicates high importance placed on risk. Individuals in state *M* may transition to state *U* due to ignoring the current risk information about the epidemic, while individuals in state *U* may transition to state *M* due to the current risk information about the epidemic. After the above definitions and calculations, the state transition probabilities in the first layer network MUM are obtained, with $g_{i}^{M}$ representing the probability of transitioning from state *M* to *U*, and $g_{i}^{U}$ representing the probability of transitioning from state *U* to *M*, described as follows:

$$g_{i}^{M}(t)=\zeta *F_{i}(t)+\left(1-\zeta \right)\cdot \left(1-r\left(z(t)\right)\right)$$ (13)

$$g_{i}^{U}(t)=\zeta *F_{i}(t)+\left(1-\zeta \right)\cdot r\left(z(t)\right)$$ (14)

where ζ represents the weight of the two influencing factors during state transition.

The second layer is the physical spreading layer of the disease, where individuals may be in one of four states: *S* (susceptible), *E* (exposed), *I* (infected), or *R* (recovered). Epidemic infected individuals are divided into *E* (exposed) and *I* (infected) states, the *E*-state refers to infected individuals without obvious clinical symptoms, while the *I*-state refers to those with obvious clinical symptoms. It is assumed that both *E*-state and *I*-state individuals can infect *S*-state individuals. *S*-state individuals become *E*-state individuals at an infection rate of β, and the infection rate for individuals in state *M* is lower than for those in state *U*, denoted as $\beta =\beta^{M}=\beta^{U}*k$, where *k* is a decay factor. *E*-state individuals then become *I*-state individuals at a probability of σ. All *I*-state individuals recover and become *R*-state individuals, acquiring immunity to the disease, at a probability of μ.

## 3 Model analysis

In this section, the Microscopic Markov chain approach (MMCA) is used to analyze and display the dynamic interaction between awareness diffusion and epidemic contagion. At time step *t*, individuals can be classified into eight different states: $p_{j}^{ME}(t),p_{j}^{MS}(t),p_{j}^{MI}(t),p_{j}^{MR}(t)$, $p_{j}^{UE}(t),p_{j}^{UI}(t),p_{j}^{US}(t),p_{j}^{UR}(t)$ represents the transition probability of individuals in the MUM layer under government control, while $g_{i}^{M}$ and $g_{i}^{U}$ represent the state transition probabilities under the behavioral game theory conditions. The specific formula expressions used are as follows:

$$w_{i}(t)=H\left[\theta -z(t)\right]m_{i}(t)$$ (15)

$$g_{i}^{M}(t)=\zeta *F_{i}(t)+\left(1-\zeta \right)\cdot \left(1-r\left(z(t)\right)\right)$$ (16)

$$g_{i}^{U}(t)=\zeta *F_{i}(t)+\left(1-\zeta \right)\cdot r\left(z(t)\right)$$ (17)

$$q_{i}^{U}(t)=\prod_{j}\left[1-b_{ij}(p_{j}^{MI}(t)+p_{j}^{UI}(t)+p_{j}^{ME}(t)+p_{j}^{UE}(t))\beta^{U}\right]$$ (18)

$$q_{i}^{M}(t)=\prod_{j}\left[1-b_{ij}(p_{j}^{MI}(t)+p_{j}^{UI}(t)+p_{j}^{ME}(t)+p_{j}^{UE}(t))\beta^{M}\right]$$ (19)

$$M_{1}=[\begin{array}{cc} 1-g_{i}^{M} & g_{i}^{M}\\ g_{i}^{U} & 1-g_{i}^{U} \end{array}],\;M_{2}=[\begin{array}{cc} 1 & 0\\ 1-w_{i}(t) & w_{i}(t) \end{array}],$$

$$M_{3}=[\begin{array}{cccc} q_{i}^{U}or\;q_{i}^{M} & 1-q_{i}^{U}or\;1-q_{i}^{M} & 0 & 0\\ 0 & 1-\sigma & \sigma & 0\\ 0 & 0 & 1-\mu & \mu \\ 0 & 0 & 0 & 1 \end{array}]$$

According to the model mechanism, The individual behavioral game state transfer probability matrix in the MUM layer is *M*_1_, the state transfer probability matrix under government intervention is *M*_2_, and the state transfer probability matrix in the epidemic layer is *M*_3_. The transition equations for each state can be obtained as follows:

$$\begin{array}{ccc} p_{i}^{MS}(t+1) & = & p_{i}^{MS}(t)g_{i}^{M}(t)q_{i}^{M}(t)\left(1-w_{i}(t)\right)+p_{i}^{MS}(t)\left(1-g_{i}^{M}(t)\right)q_{i}^{M}(t)\\ & & +p_{i}^{US}(t)\left(1-g_{i}^{U}(t)\right)q_{i}^{M}(t)\left(1-w_{i}(t)\right)+p_{i}^{US}(t)g_{i}^{U}(t)q_{i}^{M}(t) \end{array}$$ (20)

$$\begin{array}{ccc} p_{i}^{ME}(t+1) & = & p_{i}^{MS}(t)g_{i}^{M}(t)\left(1-q_{i}^{M}(t)\right)\left(1-w_{i}(t)\right)+p_{i}^{MS}(t)\left(1-g_{i}^{M}(t)\right)\left(1-q_{i}^{M}(t)\right)\\ & & +p_{i}^{US}(t)\left(1-g_{i}^{U}(t)\right)\left(1-q_{i}^{M}(t)\right)\left(1-w_{i}(t)\right)+p_{i}^{US}(t)g_{i}^{U}(t)\left(1-q_{i}^{M}(t)\right)\\ & & +p_{i}^{UE}(t)g_{i}^{U}(t)(1-\sigma )\\ & & +p_{i}^{ME}(t)\left(1-g_{i}^{M}(t)\right)(1-\sigma )+p_{i}^{UE}(t)\left(1-g_{i}^{U}(t)\right)(1-\sigma )\left(1-w_{i}(t)\right)\\ & & +p_{i}^{ME}(t)g_{i}^{M}(t)(1-\sigma )\left[1-w_{i}(t)\right] \end{array}$$ (21)

$$\begin{array}{ccc} p_{i}^{MI}(t+1) & = & p_{i}^{MI}(t)g_{i}^{M}(t)\left(1-\mu \right)\left(1-w_{i}(t)\right)+p_{i}^{MI}(t)\left(1-g_{i}^{M}(t)\right)\left(1-\mu \right)+p_{i}^{UE}(t)g_{i}^{U}(t)\sigma \\ & & +p_{i}^{UE}(t)\left(1-g_{i}^{U}(t)\right)\left(1-w_{i}(t)\right)\sigma \mathrm{\backslash notag}\\ & & +p_{i}^{UI}(t)g_{i}^{U}(t)(1-\mu )+p_{i}^{UI}(t)\left(1-g_{i}^{U}(t)\right)\left(1-w_{i}(t)\right)(1-\mu )\\ & & +p_{i}^{ME}(t)\left(1-g_{i}^{M}(t)\right)\sigma +p_{i}^{ME}(t)g_{i}^{M}(t)\sigma \left(1-w_{i}(t)\right) \end{array}$$ (22)

$$p_{i}^{US}(t+1)=p_{i}^{MS}(t)g_{i}^{M}(t)q_{i}^{U}(t)w_{i}(t)+p_{i}^{US}(t)\left(1-g_{i}^{U}(t)\right)q_{i}^{U}(t)w_{i}(t)$$ (23)

$$\begin{array}{ccc} p_{i}^{UE}(t+1) & = & p_{i}^{MS}(t)g_{i}^{M}(t)\left(1-q_{i}^{U}(t)\right)w_{i}(t)+p_{i}^{US}(t)\left(1-g_{i}^{U}(t)\right)\left(1-q_{i}^{U}(t)\right)w_{i}(t)\\ & & +p_{i}^{UE}(t)\left(1-g_{i}^{U}(t)\right)w_{i}(t)(1-\sigma )+p_{i}^{ME}(t)g_{i}^{M}(t)(1-\sigma )w_{i}(t) \end{array}$$ (24)

$$\begin{array}{ccc} p_{i}^{UI}(t+1) & = & p_{i}^{ME}(t)g_{i}^{M}(t)\sigma w_{i}(t)+p_{i}^{UE}(t)\left(1-g_{i}^{U}(t)\right)\sigma w_{i}(t)+p_{i}^{MI}(t)g_{i}^{M}(t)(1-\mu )w_{i}(t)\\ & & +p_{i}^{UI}(t)\left(1-g_{i}^{U}(t)\right)(1-\mu )w_{i}(t) \end{array}$$ (25)

$$\begin{array}{ccc} p_{i}^{UR}(t+1) & = & p_{i}^{MR}(t)g_{i}^{M}(t)w_{i}(t)+p_{i}^{UR}(t)\left(1-g_{i}^{U}(t)\right)w_{i}(t)+p_{i}^{UI}(t)\left(1-g_{i}^{U}(t)\right)w_{i}(t)\mu \\ & & +p_{i}^{MI}(t)g_{i}^{M}(t)\mu w_{i}(t) \end{array}$$ (26)

$$\begin{array}{ccc} p_{i}^{MR}(t+1) & = & p_{i}^{MI}(t)g_{i}^{M}(t)\mu \left(1-w_{i}(t)\right)+p_{i}^{MI}(t)\left(1-g_{i}^{M}(t)\right)\mu \\ & & +p_{i}^{UI}(t)\left(1-g_{i}^{U}(t)\right)\mu \left(1-w_{i}(t)\right)+p_{i}^{UI}(t)g_{i}^{U}(t)\mu \\ & & +p_{i}^{MR}(t)g_{i}^{M}(t)\left(1-w_{i}(t)\right)+p_{i}^{MR}(t)\left(1-g_{i}^{M}(t)\right)\\ & & +p_{i}^{UR}(t)\left(1-g_{i}^{U}(t)\right)\left(1-w_{i}(t)\right)+p_{i}^{UR}(t)g_{i}^{U}(t) \end{array}$$ (27)

The proportion of all states summing to 1 yields $p_{j}^{ME}(t)\;+\;p_{j}^{MS}(t)\;+\;p_{j}^{MI}(t)$ + $p_{j}^{MR}(t)$ $+\;p_{j}^{UE}(t)\;+\;p_{j}^{UI}(t)\;+\;p_{j}^{US}(t)\;+\;p_{j}^{UR}(t)=1$. When the system reaches a steady state, $p_{j}^{UI}(t\;+\;1){|}_{\left(t\to \infty \right)}\;=\;p_{j}^{UI}{|}_{\left(t\to \infty \right)}\;=\;p_{i}^{U}$. Similarly, for $p_{i}^{MS}(t\;+\;1)$, $p_{i}^{ME}(t\;+\;1)$, $p_{i}^{MI}(t\;+\;1)$, $p_{i}^{US}(t\;+\;1)$, $p_{i}^{UE}(t\;+\;1)$, $p_{i}^{UR}(t\;+\;1)$, $p_{i}^{MR}(t\;+\;1)$. Near the epidemic threshold, the probability of nodes being infected approaches zero. Assuming *S*-state individuals transition to *E*-state individuals after infection, we obtain $p_{i}^{UE}\;+\;p_{i}^{ME}\;=\;p_{i}^{E}=\in_{i}\ll 1\in_{i}$ > 0. Then, from Eqs 22 and 25, we get $p_{i}^{UI}$ + $p_{i}^{MI}=\frac{\sigma }{\mu }p_{i}^{E}$ = $\frac{\sigma }{\mu }\in_{i}$. Therefore, equations $q_{i}^{U}(t)$ and $q_{i}^{M}(t)$ can be approximated by:

$$q_{i}^{U}(t)=\underset{j}{\prod }[1-b_{ij}\left(p_{j}^{MI}(t)+p_{j}^{UI}(t)+p_{j}^{UE}(t)+p_{j}^{ME}(t)\right)\beta^{U}]\approx 1-\left(1+\frac{\sigma }{\mu }\right)\eta_{i}$$ (28)

$$q_{i}^{M}(t)=\prod_{j}\left[1-b_{ij}(p_{j}^{MI}(t)+p_{j}^{UI}(t)+p_{j}^{UE}(t)+p_{j}^{ME}(t))\beta^{M}\right]\approx 1-k\left(1+\frac{\sigma }{\mu }\right)\eta_{i}$$ (29)

where $\eta_{i}=\beta^{U}\sum_{j}\;b_{ji}\epsilon_{j}$. In the steady-state scenario, from Eqs 21 and 24, we obtain:

$$\begin{array}{ccc} p_{i}^{E} & = & p_{i}^{E}(1-\sigma )+p_{i}^{MS}(t)\left[1-q_{i}^{M}(t)+q_{i}^{M}(t)g_{i}^{M}(t)w_{i}(t)-q_{i}^{U}(t)g_{i}^{M}(t)w_{i}(t)\right]\\ & & +p_{i}^{US}(t)[1-q_{i}^{M}(t){-w_{i}(t)q}_{i}^{U}(t)+g_{i}^{U}(t)w_{i}(t)q_{i}^{U}(t)+{w_{i}(t)q}_{i}^{M}(t)-g_{i}^{U}(t)w_{i}(t)q_{i}^{M}(t)] \end{array}$$ (30)

Substituting Eqs 28 and 29 into Eq 30, we can calculate:

$$\begin{array}{ccc} \in_{i} & = & \in_{i}\left(1-\sigma \right)+p_{i}^{MS}(t)\left[k\left(1+\frac{\sigma }{\mu }\right)\eta_{i}+g_{i}^{M}(t)w_{i}(t)\left(1-k\right)\left(1+\frac{\sigma }{\mu }\right)\eta_{i}\right]\\ & & +p_{i}^{US}(t)\left[k\left(1+\frac{\sigma }{\mu }\right)\eta_{i}+g_{i}^{U}(t)w_{i}(t)\left(k-1\right)\left(1+\frac{\sigma }{\mu }\right)\eta_{i}+w_{i}(t)\left(1-k\right)\left(1+\frac{\sigma }{\mu }\right)\eta_{i}\right] \end{array}$$ (31)

Near the epidemic threshold, we have $p_{i}^{M}=p_{i}^{MS}+p_{i}^{ME}+p_{i}^{MI}+p_{i}^{MR}\approx p_{i}^{MS}p_{i}^{U}=p_{i}^{US}+p_{i}^{UE}+p_{i}^{UI}+p_{i}^{UR}\approx p_{i}^{US}$. Therefore, Eq 31 can be written as:

$$\in_{i}=\in_{i}\left(1-\sigma \right)+\left(1+\frac{\sigma }{\mu }\right)\eta_{i}\left[kp_{i}^{M}+p_{i}^{M}g_{i}^{M}w_{i}\left(1-k\right)\right]+\left(1+\frac{\sigma }{\mu }\right)\eta_{i}\left[kp_{i}^{U}+\left(g_{i}^{U}-1\right)w_{i}\left(k-1\right)p_{i}^{U}\right]$$ (32)

In the steady state, removing the $O\left(\in_{i}\right)$ terms from Eqs 20 and 23, we obtain:

$$p_{i}^{U}=p_{i}^{M}g_{i}^{M}w_{i}+p_{i}^{U}\left(1-g_{i}^{U}\right)w_{i}\;p_{i}^{M}=p_{i}^{MS}g_{i}^{M}\left(1-w_{i}\right)+p_{i}^{M}\left(1-g_{i}^{M}\right)+p_{i}^{U}\left(1-g_{i}^{U}\right)\left(1-w_{i}\right)+p_{i}^{U}g_{i}^{U}$$ (33)

Substituting Eq 33 into Eq 32:

$$\sigma \in_{i}=\left(1+\frac{\sigma }{\mu }\right)\eta_{i}\left[kp_{i}^{M}\left(1-g_{i}^{M}w_{i}\right)+(k+1)p_{i}^{U}-{\left(1-g_{i}^{U}\right)w_{i}p}_{i}^{U}k\right]$$ (34)

Simplifying Eq 34, we get:

$$\sum_{j}\left[\left(kp_{i}^{M}\left(1-g_{i}^{M}w_{i}\right)+(k+1)p_{i}^{U}-\left(1-g_{i}^{U}\right)kw_{i}p_{i}^{U}\right)b_{ji}-\frac{\mu \sigma }{\beta^{U}\left(\mu +\sigma \right)}\alpha_{ji}\right]\in_{j}=0$$ (35)

Substituting $p_{i}^{M}+p_{i}^{U}=1$ into Eq 35, we get:

$$\sum_{j}\left[\left(kp_{i}^{M}+p_{i}^{U}\right)b_{ji}-\frac{\mu \sigma }{\beta^{U}\left(\mu +\sigma \right)}\alpha_{ji}\right]\in_{j}=0$$ (36)

where $\alpha_{ij}$ is an element of the identity matrix.

Let each element of matrix C be $(kp_{i}^{M}\;+\;p_{i}^{U})b_{ji}$. Transforming the solution of Eq 36 into finding the eigenvalues of matrix C, and denoting $\Delta_{max}(C)$ as the largest eigenvalue of matrix *C*, we can obtain the epidemic threshold of this model as:

$$\beta_{c}=\frac{\mu \sigma }{\Delta_{max}(C)\left(\mu +\sigma \right)}$$ (37)

## 4 Simulation results

In this section, Monte Carlo (MC) simulation will be utilized to verify the accuracy of the MMCA analysis results, then the spread of infectious diseases and the epidemic threshold are analyzed. The two-layer network is structured as follows: An individual behavior layer and an infectious disease spreading layer. Each layer employs a BA scale-free network with 1000 nodes and an average degree of ⟨k⟩=6. The upper and lower nodes are one-to-one correspondences. The results from MMCA and MC calculations are obtained by averaging over 100 experiments. The initial state assumes that the initial proportions of individuals *U* and individuals *M* in the first layer are 100% and 0%, respectively, while the initial proportions of *S*-state and *E*-state individuals are 98% and 2%, respectively. The initial parameters are set as follows: β=0.5,k=0.4,ω=0.5,c=0.5,a=0.5,φ=0.5,η=0.5,α=1,ζ=0.5,δ=0.5,θ=0.5, σ=0.3,μ=0.5

**Fig 3:**
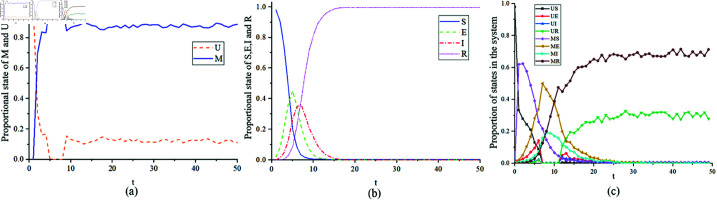
Stability of individuals in different states over time.

Fig 3 illustrates the proportions of $\rho^{M},\rho^{U},\rho^{S},\rho^{E},\rho^{I}$, and $\rho^{R}$, where $\rho^{M}=N_{M}/N$ and similarly for $\rho^{U},\rho^{S},\rho^{E},\rho^{I}$, and $\rho^{R}$. Fig 3(a) depicts the variations of individuals in different states in the MUM layer, Fig 3(b) shows the changes in individual states in the SEIR layer, while Fig 3(c) shows the evolution of the system in different states. It can be observed that after a period of time since the onset of infection, the proportion of individuals in state *E* rises for a while before rapidly declining. This is the result of government intervention in individual behavior. At almost the same time point, the proportion of individuals in state *M* becomes 1. As the epidemic situation declines, government intervention starts to decrease, leading to the emergence of individuals in state *U*. In the subsequent individual behavior game, the proportion of *M* to *U* is approximately 0.88 and 0.12, respectively.

**Fig 4:**
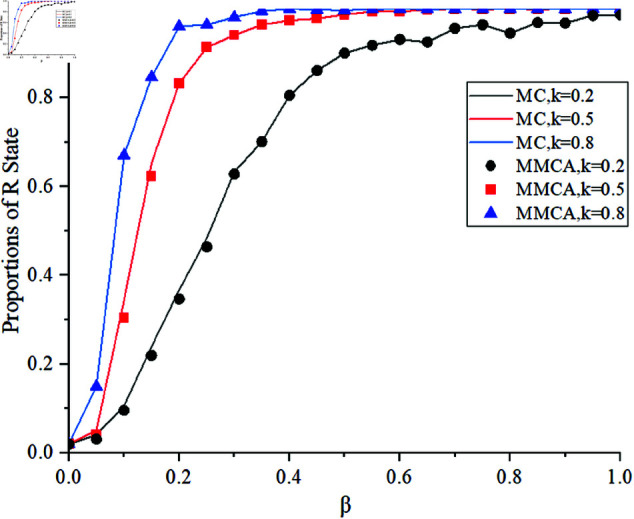
Comparison of MMCA and MC analysis results.

Fig 4 shows the comparison between the theoretical results obtained from MMCA and the simulation results obtained from MC. Different infection decay factors k = 0.2,0.5,0.8 are selected to analyze the variation of the proportion of individuals in state *R*. It can be observed that the MMCA results are in good consistency with the MC simulation results. Next, we mainly use MMCA to analyze the following results.

**Fig 5:**
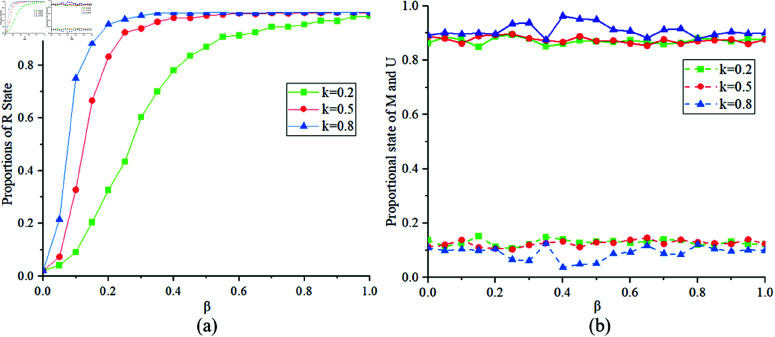
Proportions of States R, M, U at k = 0.2,0.5,0.8.

Fig 5 illustrates the proportions of states *R*, *M* and *U* at different values of k=0.2,0.5,0.8. Fig 5(a) shows the proportion of state *R* corresponding to different values of *k*. When *k* is small, according to $\beta^{M}=\beta^{U}\;*\;k$, a smaller *k* implies a lower infection rate for individuals in state *M*, resulting in slower transitions between S-E-I-R states and reducing the spread of the infectious disease, thus yielding a smaller proportion of state *R*. Fig 5(b) shows the final proportions of states *U* and *M* in the MUM layer. Conversely, when *k* is large, the spread of the infectious disease is rapid. Since the lower-layer infectious disease situation affects the state selection of upper-layer individuals, there are relatively more individuals in state *M* in the MUM layer at the final steady state.

**Fig 6:**
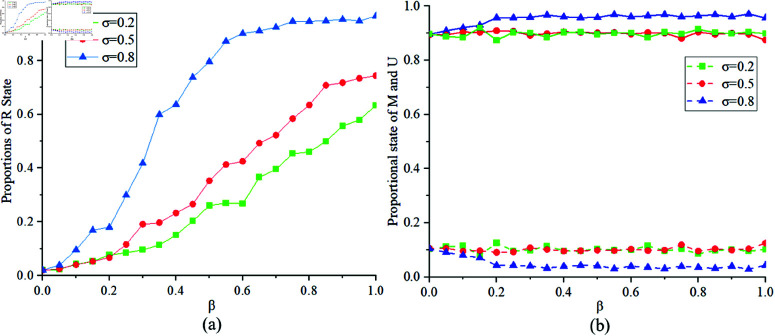
Proportions of states R, M, U at σ=0.2,0.5,0.8.

Fig 6 illustrates the proportions of states *R*, *M* and *U* at different values of σ=0.2,0.5,0.8. Fig 6(a) shows the proportion of state *R* corresponding to different values of σ. When σ is small, the transition rate from state *E* to state *I* is lower, resulting in a smaller proportion of state *R*. Fig 6(b) shows the final proportions of states *U* and *M* in the MUM layer. Similar to the variation observed with *k*, when σ changes, there is a relatively lower proportion of individuals in state *M* in the MUM layer due to the same underlying reasons.

**Fig 7:**
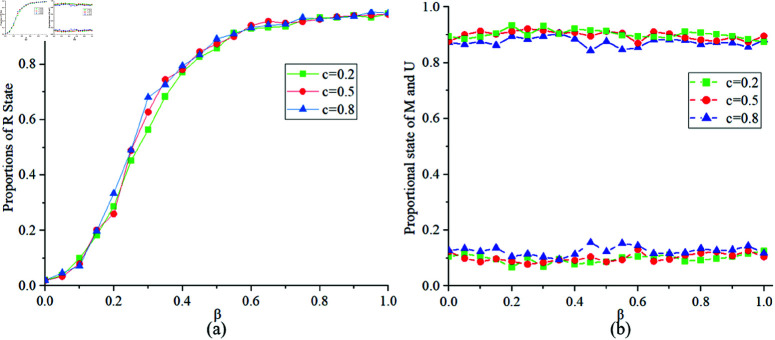
Proportions of states R, M, U at c=0.2,0.5,0.8.

Fig 7 illustrates the proportions of states *R*, *M* and *U* at different values of c=0.2,0.5,0.8. Fig 7(a) indicates that when *c* is larger, the number of infected individuals is higher, resulting in a relatively larger proportion of state *R*. Fig 7(b) indicates that when *c* is large, according to the definition of the cost function, the higher the cost of preventing infectious diseases, the smaller the proportion of individuals in state *M* in the MUM layer.

**Fig 8:**
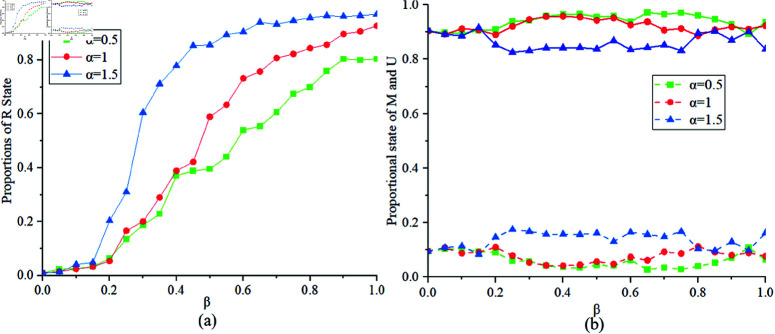
Proportions of States R, M, U at α=0.5,1,1.5.

Fig 8 illustrates the proportions of states *R*, *M* and *U* at different values of α=0.5,1,1.5. Fig 8(a) shows that when the risk perception α>1, indicating that individuals do not pay much attention to risks, the proportion of state *R* obtained is relatively large. According to the state transition formula in the MUM layer, Fig 8(b) shows that when α>1, the probability of transitioning to state *U* exceeds the probability of transitioning to state *M*. Therefore, the proportion of individuals in state *M* in the equilibrium state is smaller. Therefore, raising public awareness and encouraging individuals to take preventive measures against epidemics can help suppress the spread of the disease.

**Fig 9:**
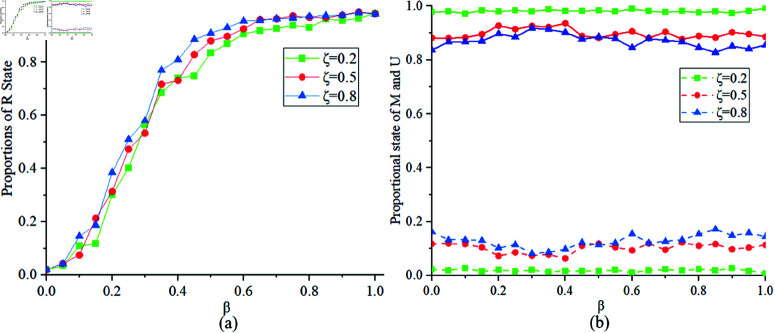
Proportions of states R, M, U at ζ=0.2,0.5,0.8.

Fig 9 illustrates the proportions of states *R*, *M* and *U* at different values of ζ=0.2,0.5,0.8. Fig 9(a) indicates that when ζ is smaller, the proportion of state *R* obtained is smaller. Fig 9(b) suggests that when ζ is smaller, there are more individuals in state *M* in the MUM layer. According to the modeling formula, this may be because the game behavior plays a more important role in the MUM layer. Therefore, lowering the cost of individual protective measures can increase people’s acceptance of such measures, thereby reducing the chances of infection and the spread of epidemics.

**Fig 10:**
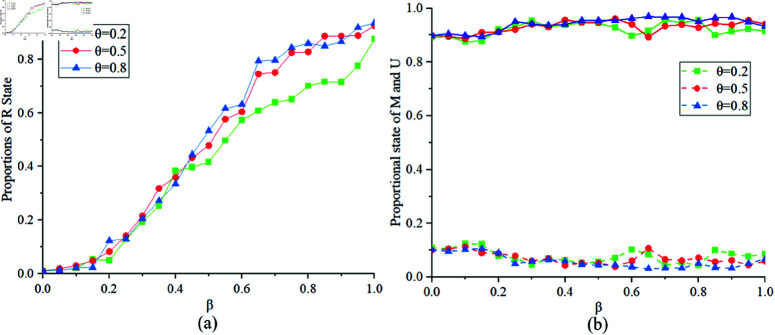
Proportions of states R, M, U at θ=0.2,0.5,0.8.

Fig 10 illustrates the proportions of states *R*, *M* and *U* at different values of θ=0.2,0.5,0.8. Fig 10(a) indicates that when the government control threshold θ is smaller, the final proportion of state *R* is smaller. This suggests that when θ is small, the number of susceptible individuals transitioning to exposed individuals decreases, resulting in fewer individuals transitioning to other states. Fig 10(b) shows that, for the same reasons as with changes in the value of *k*, there are slightly fewer individuals in state *M* in the MUM layer. Therefore, the government’s establishment of a reasonable epidemic control threshold is beneficial for controlling the spread of infectious diseases.

**Fig 11:**
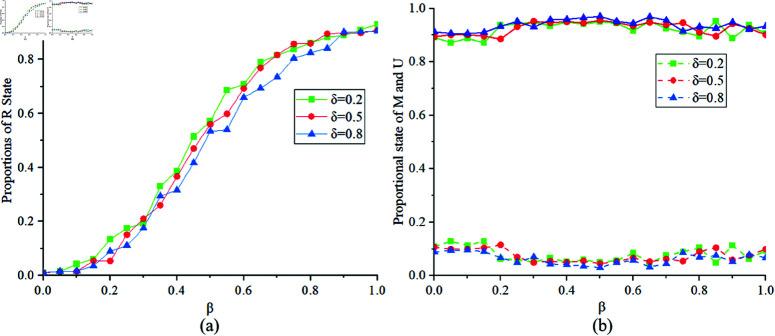
Proportions of States R, M, U at δ=0.2,0.5,0.8.

Fig 11 illustrates the proportions of states *R*, *M* and *U* at different values of δ=0.2,0.5,0.8. Fig 11(a) indicates that when the government control intensity δ is large, the proportion of state *R* obtained is smaller. Fig 11(b) shows that, according to the modeling mechanism, there are more individuals in state *M* in the MUM layer. Therefore, strengthening the government’s regulation of individual protective behaviors during epidemic outbreaks and ensuring that the public adopts effective protective measures can help reduce the final infection scale.

**Fig 12:**
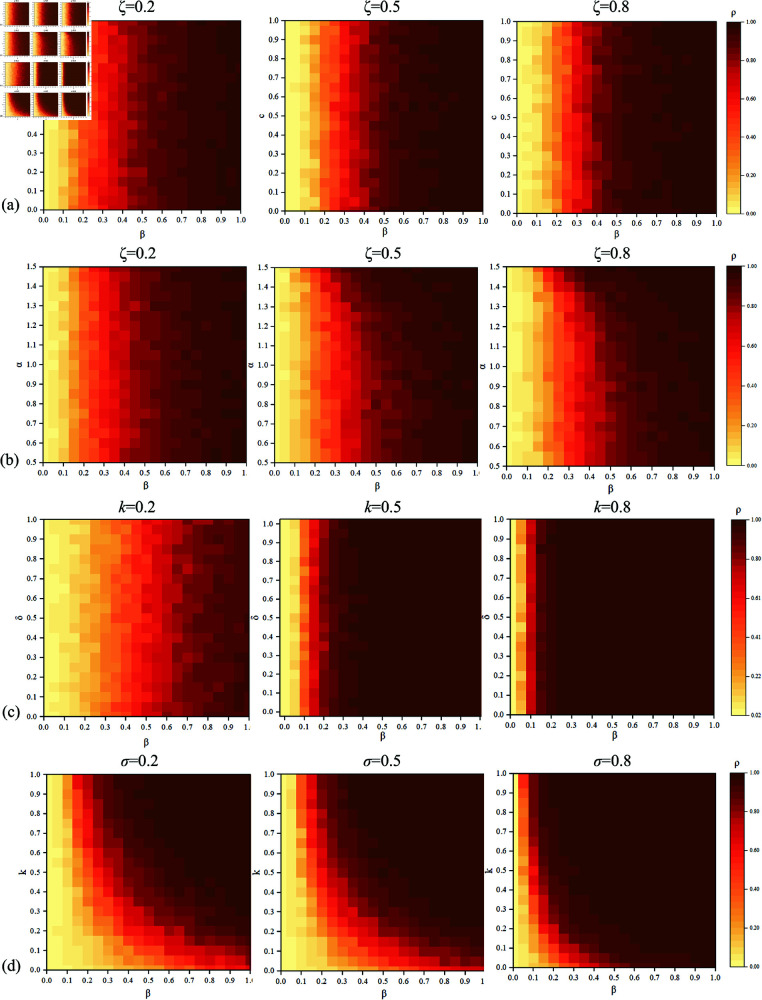
Phase Diagrams of R-state Proportions under Different ζ, k, σ.

This study examines the phase diagrams of *R*-state proportions corresponding to different parameters in the model, where shades of red indicate larger proportions of the *R*-state within a 20×20 grid at steady state. Fig 12(a) illustrates the phase diagrams of β and *c* when ζ=0.2,0.5,0.8. When ζ is larger, the proportion of state *R* is greater. Similarly, when *c* is larger, the proportion of state *R* is also greater. Fig 12(b) shows the phase diagrams of β and α, when ζ=0.2,0.5,0.8. An increase in α indicates individuals disregarding risks, leading to a larger proportion of the *R*-state. Fig 12(c) presents the phase diagrams of β and δ, when k=0.2,0.5,0.8. It can be observed that the proportion of the *R*-state decreases with decreasing *k*, while it decreases with increasing δ. Fig 12(d) exhibits the phase diagrams of β and *k* when σ=0.2,0.5,0.8. As σ increases, the proportion of the R-state increases, and similarly, it increases with increasing *k*. These findings are consistent with previous observations.

**Fig 13:**
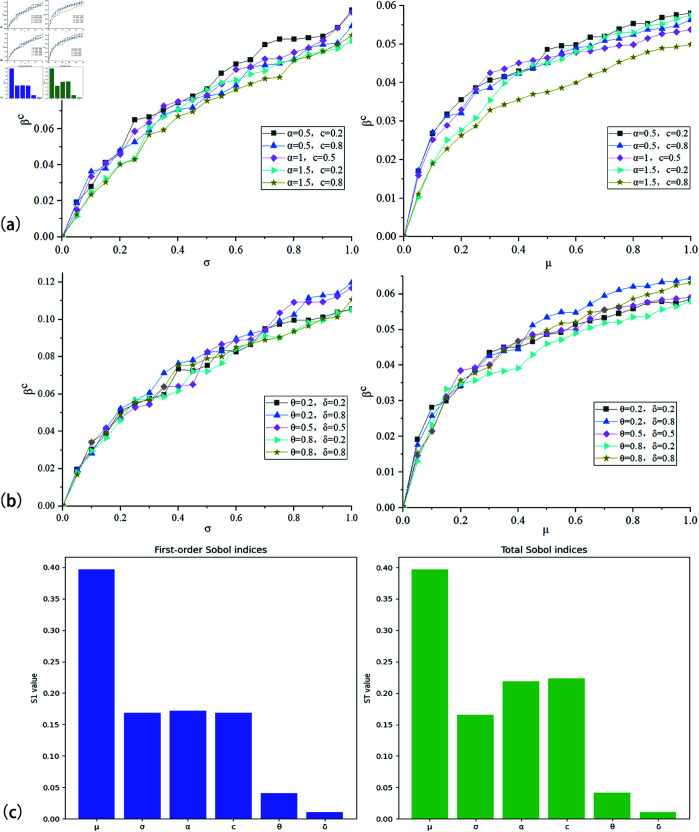
Corresponding $\beta^{c}$ under different α,c,σ,θ parameters.

This paper also investigates the influence of different parameters in the model on the infectious disease threshold. As seen in Fig 13, the trend indicates that as σ increases, the threshold increases, and as μ increases, the threshold also increases. From Fig 13(a), it is evident that as c increases, the threshold decreases, and as α increases, the threshold decreases. This indicates that reducing individual protective costs and increasing individuals’ perception of epidemic information will inhibit the spread of infectious diseases. From Fig 13(b), it can be seen that as θ increases, the threshold decreases, and as δ increases, the threshold increases. This suggests that setting reasonable risk warning thresholds and strengthening individual control during the spreading process at the government level are beneficial for suppressing the spread of infectious diseases. We conducted a sensitivity analysis of the outbreak threshold *β*^c^ with respect to certain parameters in the model. As shown in Fig 13(c), it can be observed that the recovery rate, epidemic risk perception, individual protective behavior costs, and other factors have a significant impact on the outbreak threshold.

## 5 Conclusion

Infectious diseases have various harmful effects, impacting health, economy, society, environment, and global stability. Although the government can effectively control the spread of infectious diseases by issuing and formulating effective prevention and control strategies, the public may not be able to fully comply with infectious disease prevention measures for various reasons. Therefore, this paper establishes a two-layer coupled network model to study the dynamic interaction between individual protective behavior and SEIR-based infectious disease spreading, incorporating government intervention in individual behavior into the two-layer network model, a threshold model is also introduced to control individual protective behavior based on the epidemic situation. The results show that reducing personal protective costs, increasing individual attention to disease information, timely adjustments of government control measures according to the current epidemic situation, and strengthening government control over infectious diseases can effectively raise the outbreak threshold of infectious diseases. In the future, closer to real situations, the dynamic interaction between game theory under various behavioral states and infectious disease spreading can be considered, or the higher-order structure of the network can be integrated into the game behavior.

## Supporting information

Data (ZIP)
